# Gastrocnemius muscle flap for coverage of knee defects in the injuries of popliteal artery: a clinical case report

**DOI:** 10.3389/fsurg.2024.1490493

**Published:** 2024-12-24

**Authors:** Mohammadali Babaei Zarch, Samira Mahmoudi, Mohammad Hadi Gerami Shirazi, Armin Fereidouni

**Affiliations:** ^1^ Orthopedic & Rehabilitation Research Center, Shiraz University of Medical Sciences, Shiraz, Iran; ^2^ Faculty Member, Department of Anesthesia, School of Nursing and Midwifery, Shiraz University of Medical Sciences, Shiraz, Iran; ^3^ Department of Operating Room Technology, Community Based Psychiatric Care Research Center, School of Nursing and Midwifery, Shiraz University of Medical Sciences, Shiraz, Iran

**Keywords:** gastrocnemius flap, popliteal artery repair, knee defects, popliteal artery, flap

## Abstract

The use of the gastrocnemius muscle flap has become an excellent choice for coverage of Knee Defects. However, the surgical management of gastrocnemius muscle flap in the injuries of the popliteal artery remains a challenging therapeutic problem. The purpose of this manuscript is to present a case of a successful knee gastrocnemius flap in a patient with popliteal artery injuries. In 2024, a 46-year-old woman with a tibia fracture and popliteal artery injury went to the emergency department of Namazi Hospital. In the first step, the external fixator of the tibia bone was performed to fix the fracture. Then, popliteal artery anastomosis was performed. After two weeks, all internal implants were removed due to abscess. Four weeks later, the patient's skin developed necrosis and was repaired using medial hemi gastrocnemius Muscle flaps. We present a case of the successful use of medial hemi gastrocnemius Muscle flaps for Coverage of knee defects in the injuries of the popliteal artery.

## 1 Introduction

Reconstruction of defects caused by trauma and open fractures is vital for the injured person in terms of aesthetics, treatment, and psychosocial issues (1). These defects can be caused by tumors, infections, or trauma, which, if left untreated, lead to pain, limited movement, and apparent dissatisfaction in people (2, 3). Since 1978, the gastrocnemius muscle flap (GMF) has been introduced as a reliable and safe method for the reconstruction of soft tissue defects of the knee due to its good blood supply (4). In general, the gastrocnemius muscle flap is performed in two ways, external and internal, as a myocutaneous flap, usually when the soft tissue defect is so large that the patient's tendon and bone are visible or the surgical incision is so deep that the two edges of the tissue are not closed (5, 6).

The transfer of one head of the gastrocnemius muscle in the flap technique not only does not lead to the dysfunction of the donor organ but also leads to the improvement of the function of the recipient organ and the return of its beauty (3). Other advantages of this technique include a high success rate, easy removal with minimal complications in the donor tissue, capacity to regenerate the removed tissue, and reducing the possibility of infection (2, 6). The gastrocnemius muscle is supplied with blood by the medial and lateral sural arteries and direct branches of the popliteal arteries (6).

The sural arteries are large vessels that arise on each side of the popliteal artery to provide a vascular supply to the gastrocnemius, soleus, and plantaris muscles. Both the medial and lateral heads of gastrocnemius are supplied by the lateral and medial sural arteries, which are direct branches of the popliteal artery (7). This good source of blood supply, by helping to regenerate the muscle, makes the GMF an effective treatment method for large defects and traumas (2). An injury to the sural and popliteal vessels following trauma in the knee area disrupts the effectiveness of the GMF technique and is considered a contraindication for the use of this technique (5). This manuscript aims to present a case study of GMF for the restoration of a tissue defect in the knee area caused by a popliteal artery injury.

## 2 Case description

A 46-year-old female was brought to the emergency department of Namazi Hospital (the largest medical center in the south of Iran) because of a car accident. The patient's left leg had an open fracture of the tibia plateau type 6 Schatzker or type 5 Hohl and more classifications along with popliteal artery injury and pulseless. *The injury is only in the intima layer of the popliteal artery, but there is no injury to the popliteal vein*. The patient's open fracture was type IIIc according to the Gastilo-Anderson classification and had significant loss of tissue with an associated vascular injury.

### 2.1 Treatment

**Figure 1:**
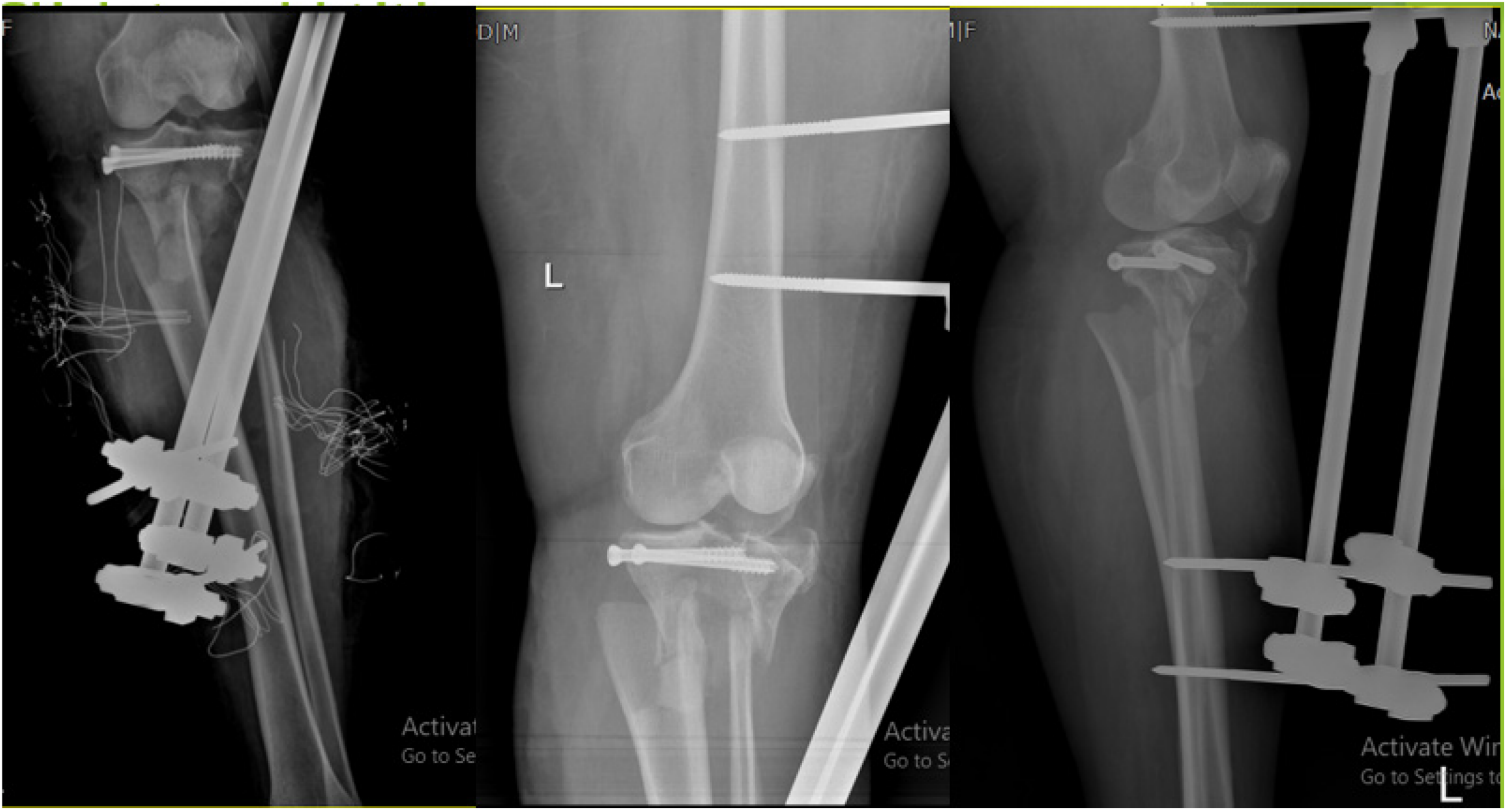
Use of external fixator and cancellous screw for temporary fracture fixation.

In the first step, *an* external fixator (5-degree knee flexion position) of the tibial along with 2 cancellous screws (close method) *was* performed to fix the fracture area in the orthopedic service. The use of screws was for the temporary reconstruction of the articular surface (Figure 1).

**Figure 2:**
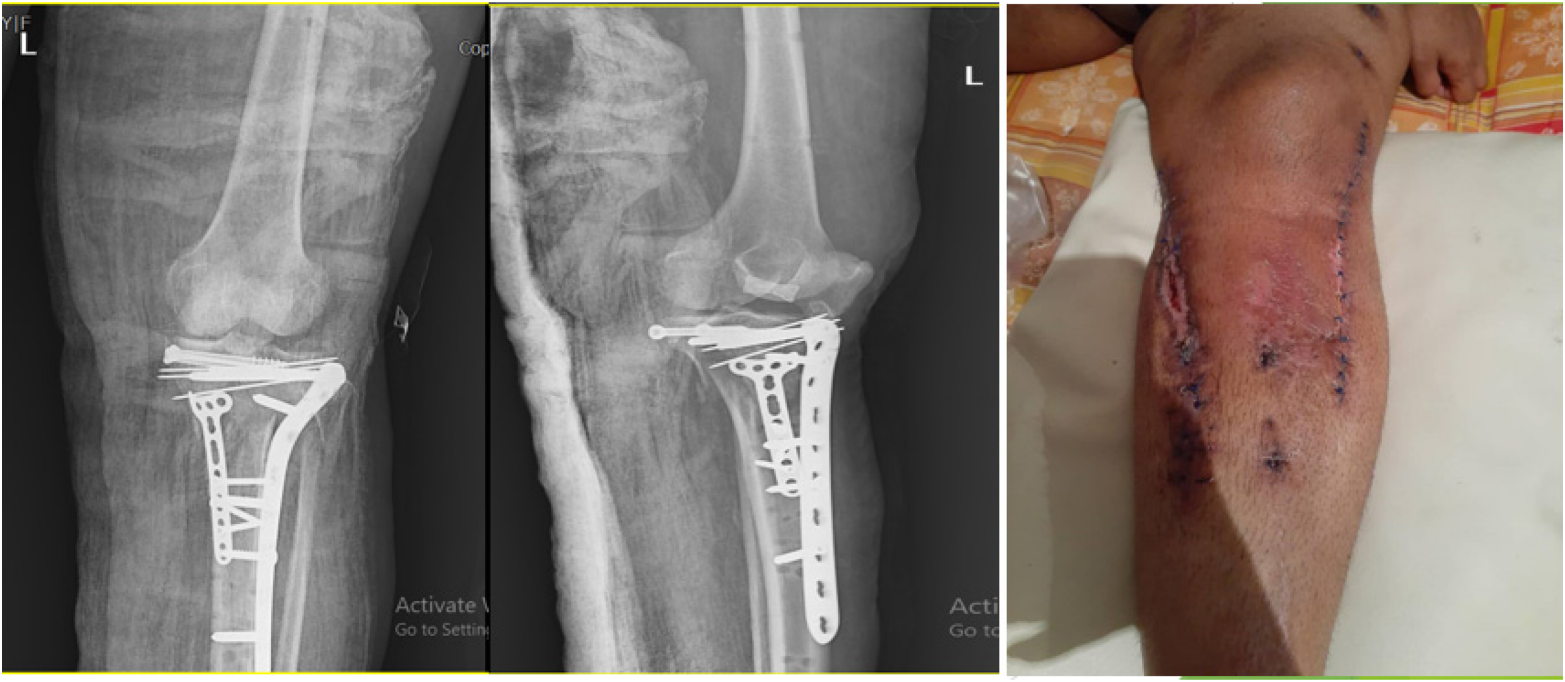
Four weeks after popliteal artery anastomosis and placement of internal implants.

In the next step, popliteal artery anastomosis was performed about 20 min after the temporary fixation of the fracture in the vascular surgery department through a medial knee incision. The vascular surgery team checked the blood flow of the anastomosed vessels by CT angiography after the surgery. The patient was discharged from the hospital, and after 6 weeks, when the condition of the skin and soft tissue improved, the external fixator was removed from the operating room. After 5 days of using a lateral approach, the patient's fracture was repaired using a proximal lateral tibia locking plate, and a buttress plate was fixed (Figure 2).

After 2 weeks of surgery, an abscess between the medial incision (for popliteal artery repair) and lateral incision (for fracture fixation) with bone involvement was diagnosed by an orthopedic doctor. Methicillin-resistant staphylococcus aureus (MRSA) type of infection was diagnosed by sending the culture from surgical site infection. At this step, due to the spread of the infection deep into the bone during surgery, all internal implants (plates and screws) were removed. After extensive washing of the surgical site, several steps in consecutive weeks, and control of the infection (negative result of culture (SSI), the Masquelet technique was performed on the patient. This technique involves the insertion of an antibiotic-loaded polymethylmethacrylate (PMMA) bone cement spacer into the area of bone loss. The bone is stabilized by external fixation. The cement spacer stimulates a biological membrane to form around it (8).

Four weeks after this technique, the patient's skin was necrotic in an area of 4 cm × 10 cm, and the patient's bone was exposed.

**Figure 3:**
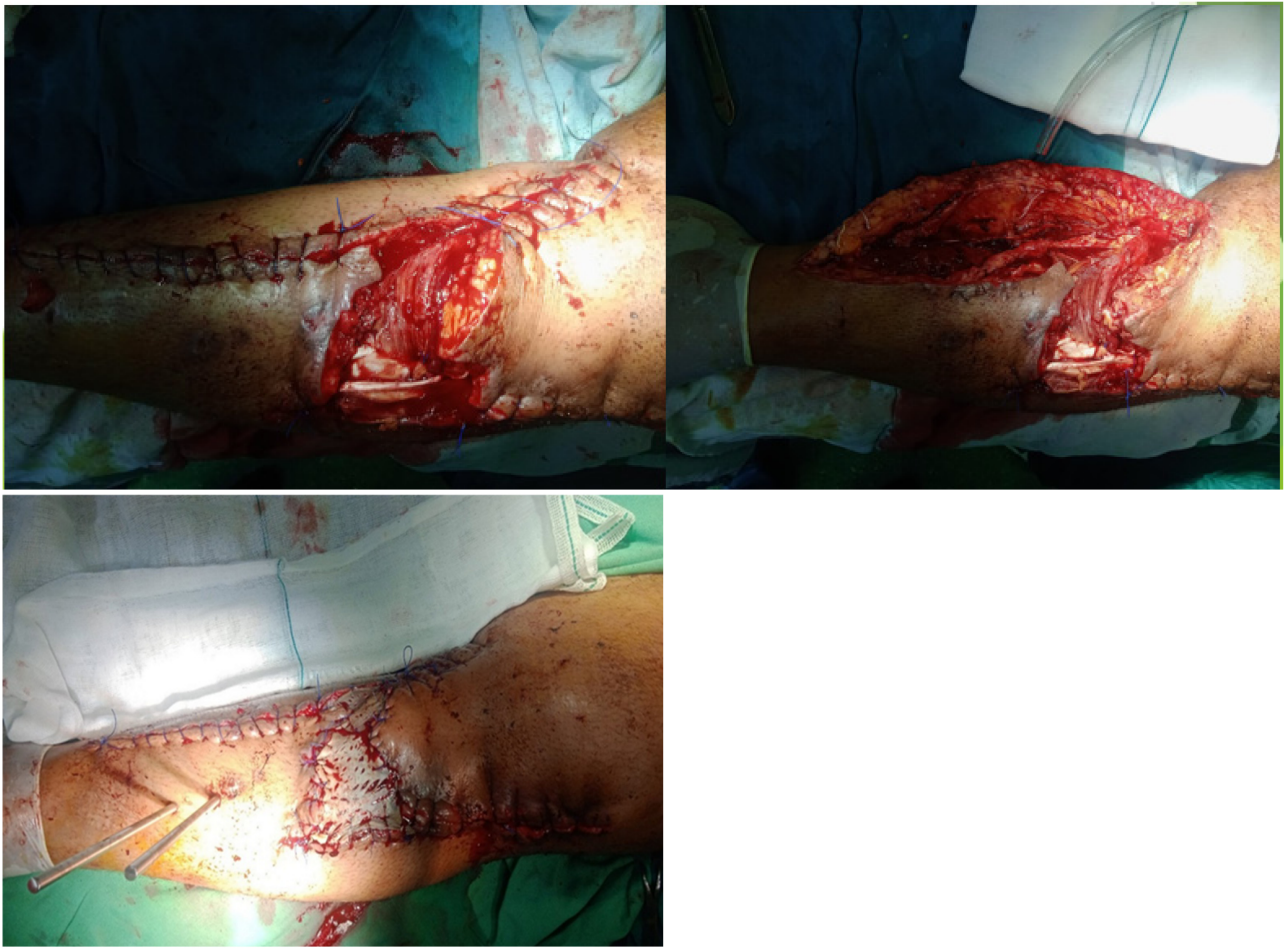
Medial hemi gastrocnemius flap and split thickness skin graft.

**Figure 4:**
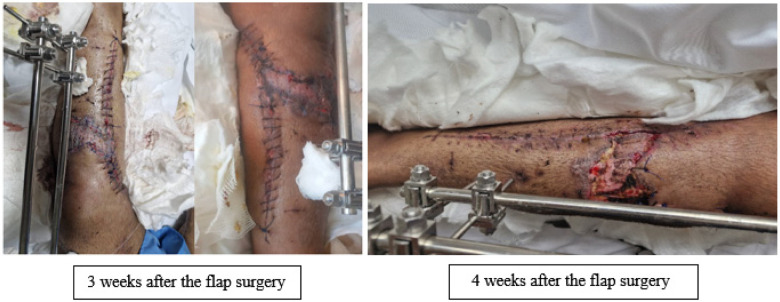
Repaired flap after 3 and 4 weeks.

**Figure 5:**
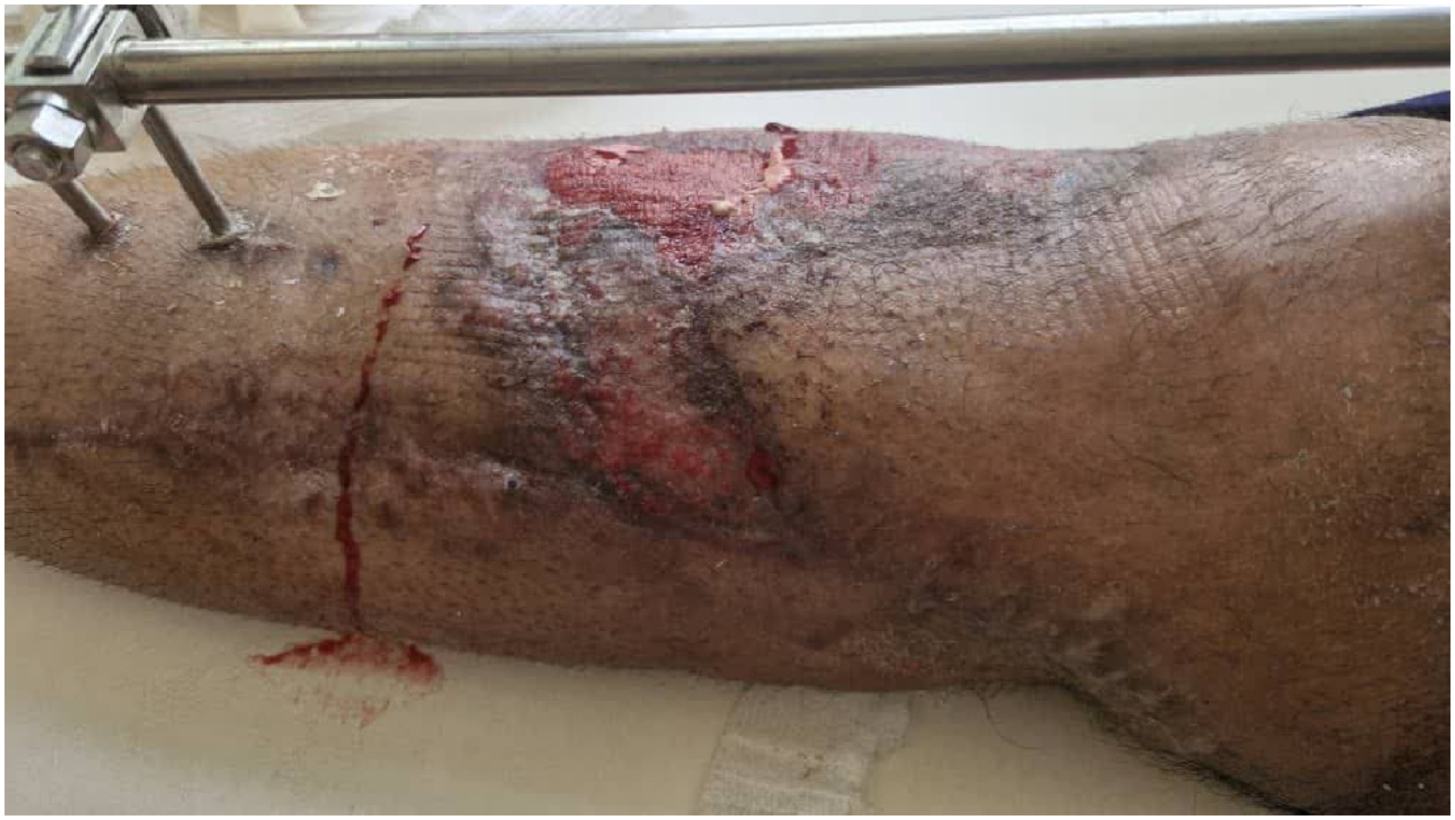
Repaired flap after 8 weeks.

**Figure 6:**
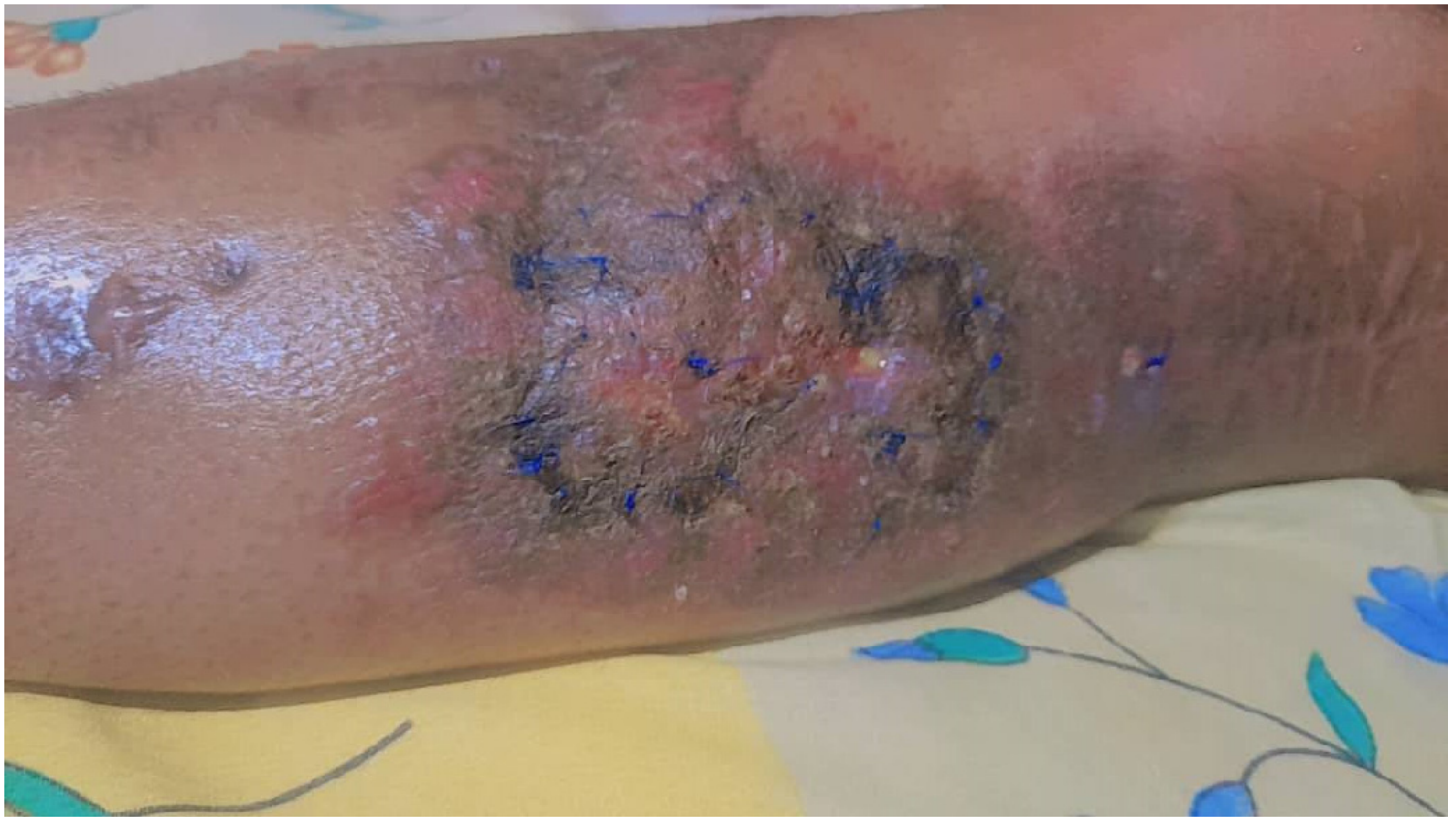
Repaired flap after 12 weeks.

At this step, the patient's skin was repaired using MHGMF (based on sural vascularity), and a split-thickness skin graft was performed (Figure 3). Before the flap surgery, the blood flow of the anastomosed vessels was checked through CT angiography. Also, the patient's lower limb pulse and popliteal pulse were checked by physical exam. After 4 weeks, the flap is completely repaired (Figures 4–6). Also, negative pressure wound therapy was used as an Alternative treatment for soft tissue coverage during infection until its flapping.

Surgical Approach for Medial gastrocnemius Flap:

- longitudinal incision that Incision in the posteromedial part of the shank, from the tibial plateau to a point 10 cm above the ankle;
- -if the flap is to be tunneled under a skin bridge, the incision should be placed even more posteriorly, to ensure adequate skin bridge;
- -The saphenous vein is left intact;
- gastrocnemius is separated from the overlying sub *Q* tissue;
- avascular plane is developed between the medial head of the gastrocnemius and soleus;
- at the median raphe, small vessels may be identified between the gastrocnemius and soleus;
- median raphe:
- at times it is difficult to identify the midline raphe between the medial and lateral head of the gastrocnemius;
- The gastrocnemius muscle is composed of two heads that are partially fused in the midline of the calf, but more proximally the two heads can be bluntly separated (at the popliteal fossa);
- proximally sural nerve traverses the midline between the two heads, but then passes lateral to the raphe;
- distally the attachment of the medial head is released w/ a small portion of the Achilles tendon attached to the flap;
- after its attachment to the Achilles tendon is freed, the neurovascular pedicle is isolated;
- fascia on the deep surface of the muscle can be scored longitudinally to increase the breadth of the flap;
- if indicated, the pedicle can be dissected to its origin and the muscle's femoral attachments divided, w/ care to avoid sural artery damage

The contraindications for using medial hemi gastrocnemius are active infection, repair of popliteal artery, and popliteal aneurysm (5, 9). In this case, despite the injury of the popliteal artery, the flap was repaired well. To complete the fixation of the patient's fracture at this step, a bone autograft graft [the tissue typically comes from the top of the hip bone (the iliac crest)] was used and placed instead of bone cement at the fracture site followed by external and final fixation with ilizarov external fixator.

### 2.2 Ethical considerations

All named authors meet the International Committee of Medical Journal Editors (ICMJE) criteria for authorship for this article, take responsibility for the integrity of the work, and approve this version to be published. Written informed consent was obtained from the patient for publication of this case report and accompanying images.

## 3 Discussion

The gastrocnemius muscle is the most superficial muscle of the posterior calf. It has two heads, medial and lateral, which form the distal border of the popliteal fossa. Each head can be used as a separate muscle or musculocutaneous unit, based on its pedicle. The medial head originates from the medial condyle of the femur, and the lateral from the lateral condyle of the femur. Both heads are inserted into the calcaneus through the Achilles tendon. The gastrocnemius muscle helps the plantar flexion of the foot. One or both heads of the muscle are expandable if the soleus muscle is intact (7).

Reconstruction of defects caused by tibial bone fractures along with popliteal artery damage is one of the most important and challenging issues. It is necessary to use restorative procedures to cover the bones or joints and prevent infection. There are several methods for repairing damaged areas, one of which is the use of the gastrocnemius flap technique (10). The gastrocnemius flap is the primary muscle flap used in the reconstruction of the upper third of the leg (11).

The suitability of the gastrocnemius flap technique has been investigated and confirmed in various studies. For example, in a study, Mayoly et al. used the gastrocnemius flap technique to reconstruct knee defects in injured patients following trauma, tumors, and knee replacements. All patients had a uncomplicated recovery after 15 days (6).

Shahzad et al. investigated the results of the gastrocnemius flap technique on 139 patients with soft tissue defects in the leg. The results after 6 weeks showed that more than 95% of the patients achieved remission (3). Contrary to the present study, Seo et al. conducted a study aimed at analyzing flap failure in tissue with anastomosed vessels. The study was conducted on 5 flaps (including 3 free radial forearm flaps, 1 free latissimus dorsi flap, and 1 free fibula flap). The results of the study reported the cause of failure in 5 flaps as the presence of vascular thrombosis and vascular endothelium tissue damage following anastomosis (12). Casey et al. conducted a study with the aim of investigating the outcomes of flap healing following injury and revascularization. In line with the present study, the researchers did not report a significant difference in the outcomes of the flap with anastomosis and revascularization with the flap without revascularization (13).The health of the blood vessels of the gastrocnemius muscle is necessary to perform the gastrocnemius flap. For this reason, it is recommended to perform arteriography before using the flap. Patients who need gastrocnemius flap with complications such as active infection, repair of popliteal artery, and popliteal anorism will suffer from a disorder in the recovery process after the flap due to insufficient blood supply to the muscle (5). Therefore, according to medical science, the use of gastrocnemius flap is considered a contraindication for these patients. Nevertheless, in the present study, the gastrocnemius flap procedure was performed in a patient with popliteal artery injury without any complications, and the results were successful.

## 4 Conclusions

The use of the gastrocnemius muscle flap to cover soft tissue defects of the knee can be successful even in patients with popliteal artery repair. It is suggested to carry out more research in order to know the factors affecting the success of this technique and finally to add the gastrocnemius flap as a selective technique in people with leg tissue defects along with popliteal artery repair.

## Data Availability

The original contributions presented in the study are included in the article/Supplementary Material, further inquiries can be directed to the corresponding author.
